# Hearing performance and voice acoustics of cochlear implanted children^☆^^☆☆^

**DOI:** 10.1016/j.bjorl.2015.11.002

**Published:** 2015-12-02

**Authors:** Ana Cristina Coelho, Alcione Ghedino Brasolotto, Maria Cecília Bevilacqua, Adriane Lima Mortari Moret, Fayez Bahmad Júnior

**Affiliations:** a Hospital Universitário de Brasília (HUB/UnB), Brasília, DF, Brazil; b Department of Speech, Language and Hearing Sciences, Faculdade de Odontologia de Bauru, Universidade de São Paulo (USP), Bauru, SP, Brazil; c Post-Graduate Program in Health Sciences, Universidade de Brasília (UnB), Brasília, DF, Brazil

**Keywords:** Voice, Voice quality, Cochlear implant, Voz, Qualidade da voz, Implante coclear

## Abstract

**Introduction:**

The voice of hearing-impaired individuals has been described extensively, and exhibits abnormalities in quality, articulation and resonance. Having an understanding of the aspects that may have an impact on voice characteristics of cochlear implant users is important for users and for professionals in this field.

**Objective:**

To verify the existence of correlation between age, time of device use, voice detection threshold, hearing category score and language category score with acoustic data of voices of cochlear implanted children.

**Methods:**

Retrospective study. Fifty-one children ranging in age from 3 years to 5 years and 11 months who unilaterally used cochlear implants participated. Acoustic analysis of the sustained vowel/a/, sequential speech and spontaneous speech was performed. The results were correlated with demographic data and hearing test results.

**Results:**

Children with worse voice detection threshold showed higher frequency in the sustained vowel (*p* ≤ 0.001) and in the spontaneous speech (*p* ≤ 0.005).

**Conclusion:**

There was a correlation between the voice detection threshold and the frequency values of the sustained vowel and spontaneous speech of the studied population.

## Resumo

**Introdução:**

A voz do indivíduo com deficiência auditiva tem sido amplamente caracterizada, estando comprometida em termos de tipo de voz, articulação e ressonância, sendo que o conhecimento dos aspectos que possam ter impacto nas características vocais de usuários de implante coclear é de suma importância para os usuários e profissionais da área.

**Objetivo:**

Verificar a existência de correlação entre idade, tempo de uso, limiar de detecção de voz, escore da categoria de audição e escore da categoria de linguagem com dados acústicos de vozes de crianças com implante coclear.

**Método:**

Estudo retrospectivo. Participaram 51 crianças usuárias de implante coclear com idade de 3–5 anos e 11 meses. Foi realizada análise acústica da vogal sustentada/a/, fala encadeada e conversa espontânea. Os resultados foram correlacionados com dados demográficos e resultados de testes auditivos.

**Resultados:**

Crianças com pior desempenho no teste de detecção de voz apresentam voz mais aguda na vogal sustentada (*p* ≤ 0,001) e na conversa espontânea (*p* ≤ 0,005).

**Conclusão:**

Houve correlação entre os limiares de detecção de voz e os valores de frequência na vogal sustentada e conversa espontânea na população estudada.

## Introduction

The vocal behavior of children can vary considerably, and parameters such as resonance, pitch and loudness are essential for determining this performance and to identify voice abnormalities.^1^ The expected vocal standard in childhood includes fundamental frequency above 250 Hz, high pitch, moderate to high intensity and abrupt vocal attack. Slight nasality, hoarseness and breathiness can be observed.^2^

In the child with hearing impairment, especially if pre-lingual, there is a flaw in auditory monitoring of the voice resulting in several deviations of vocal production, because in addition to neuromuscular control, hearing is important for good performance in oral communication. The auditory system is able to regulate voice parameters such as intensity, extension and frequency.^3^, ^4^ Among other characteristics, the most common vocal disorders in hearing impaired individuals include unpleasant quality, strain, resonance imbalance, high frequency, altered breathing pattern and utterance with excessive variation.^5^, ^6^, ^7^

Especially when performed early in life, the cochlear implant is a most important advancement in the treatment of children with pre-lingual hearing impairment, and implanted children achieve better auditory perception of speech sounds, incidental appropriation of oral language, and better speech intelligibility and vocal production.^8^, ^9^ However, despite the marked improvements after the implant, it is possible that users of such devices show a suboptimal voice quality.^10^ Therefore, understanding the physiological processes involved in voice control of CI users is a major challenge for the specialists working in this area.^11^

It is known that the results with cochlear implants are closely related to the age at which the child received the device.^12^ Additionally, follow-up of these children monitors the performance of hearing, language and speech in clinical and daily contexts, and the performance evaluation supplies data to define more accurately the hearing ability of each child,^4^ as well as their oral communication with respect to voice and speech.

In this study we question whether the vocal characteristics of cochlear implanted children are related to other demographic and hearing performance characteristics. Therefore, the aim of this study was to investigate the correlation between age, time of device use, voice detection threshold, hearing category score^13^ and language category score^14^ and acoustic data of voices of cochlear implanted children.

## Method

This study was approved by the Ethics Committee in Research with Human Beings of the institution where it was held, under process N. 131/2010. All parents/guardians signed the free and informed consent form.

Fifty-one children with pre-lingual hearing impairment, who unilaterally used the *Nucleus* 24 *Contour*, *Nucleus Freedom Contour*, Pulsar_CI_^100^ or Sonata_TI_^100^ cochlear implants, participated in the study.

The inclusion criteria for participation in the study were severe or profound bilateral congenital sensorineural hearing loss, no intellectual or emotional impairments, participation in an auditory habilitation program in the city of origin, cochlear implantation before 36 months of age with full insertion of the electrodes and use of the CI for more than a year.

Considering the purpose of the study, speech samples were collected from participants, comprising the utterance of the vowel/a/three times, sequential speech (counting from 1 to 5) and spontaneous speech for subsequent acoustic analysis. The recording program used was Sony Sound Forge (Sony Pictures Digital Inc 8.0) with sampling rate of 44,100 Hz, 16 Bit, mono channel. The participant remained seated during the recording in an acoustically treated audiometric testing room. The sound card M-Audio Fast Track Pro and headset microphone AKG C512 positioned at 45° and with a distance of 3 cm from the mouth were used.

Acoustic analysis was performed with the Multi Dimensional Voice Program, model 5105, version 2.5.2 (Kay Elemetrics), and Real Time Pitch, model 5121, version 2.5.2 (Kay Elemetrics). The selected acoustic parameters for analysis of the long vowel were:

Frequency measurements: fundamental frequency mean (*f*_0_) and standard deviation;
Long-term and short-term frequency disturbance measures: coefficient of variation of *f*_0_ (v*f*_0_) and jitter;
Long-term and short-term amplitude disturbance measures: coefficient of variation of the amplitude (vAm) and shimmer;
Noise measurements: vocal turbulence index (VTI), soft phonation index (SPI), harmonics-to-noise ratio (HNR) and degree of sub-harmonics (DSH).

The selected parameters for analysis of sequential speech and spontaneous speech samples were frequency measurements: frequency mean, frequency extension measured in Hertz (Hz), minimum frequency, maximum frequency, frequency standard deviation and frequency extension measured in semitones.

Data regarding age, time of cochlear implant use, free-field voice detection threshold (VDT), hearing category score and language category score were collected on the same day of the voice recording, during routine evaluations by speech therapists from the cochlear implant service of the institution where the study was carried out.

The selected variables are continuous and normally distributed. Therefore, the statistical test used for the analysis was Pearson's correlation with a 5% significance level. A classification scale was used, where the correlation of 0–20% is considered poor, 20–40% bad, 40–60% regular, 60–80% good and 80–100% excellent.

## Results

This study found a correlation between age, time of device use, voice detection threshold, hearing category score and language category score with acoustic data of voices of cochlear implanted children. The data that characterize the study population are shown in Table 1. The mean results of the acoustic analysis of voice signals of the participants are shown in Table 2. Because not all children were able to perform the three utterances, there was a slight variation in the total number of each utterance.

**Table 1:** Characteristics of the sample regarding the assessed data.

Data	Mean (±standard deviation)
Age (months)	48.38 (±8.01)
Time of cochlear implant use (months)	24 (±8.03)
Voice detection threshold (dB)	24.23 (±4.94)
Hearing category	3.55 (±1.3)
Language category	3.14 (±1.8)

SD, standard deviation; dB, decibel.

**Table 2:** Mean and standard deviation of the acoustic data of the sustained vowel (*n* = 46), sequential speech (*n* = 45) and spontaneous speech (*n* = 47).

Parameter	Sustained vowel Mean (±SD)	Sequential speech Mean (±SD)	Spontaneous speech Mean (±SD)
Mean *f*_0_ (Hz)	293.53 (±55.5)	–	–
SD *f* (Hz)	7.78 (±4.08)	–	–
v*f*_0_ (%)	278 (±1.48)	–	–
Jitter (%)	1.3 (±1.02)	–	–
vAm (%)	16.09 (±5.84)	–	–
Shimmer (%)	3.19 (±1.17)	–	–
VTI (%)	0.07 (±0.07)	–	–
SPI (%)	5.61 (±4.69)	–	–
NHR (%)	0.13 (±0.02)	–	–
DSH (%)	2.16 (±3.12)	–	–
Mean Freq (Hz)	–	296.14 (±24.39)	304.70 (±31.10)
Extension (Hz)	–	165.83 (±50.35)	168.32 (±43.72)
Min Freq (Hz)	–	213.24 (±24.87)	209.20 (±27.47)
Max Freq (Hz)	–	372.23 (±28.99)	377.54 (±33.25)
SD Freq (Hz)	–	34.04 (±15.27)	38 (±15.99)
Extension (semitones)	–	9.69 (±2.79)	10.19 (±2.67)

Mean *f*_0_, mean of the fundamental frequency; SD *f*_0_, standard deviation of the fundamental frequency; v*f*_0_, coefficient of variation of *f*_0_, coefficient of amplitude variation; VTI, voice turbulence index; SPI, soft phonation index; HNR, harmonics-to-noise ratio; DSH, degree of sub-harmonics; Mean Freq, mean frequency; Min Freq, minimum frequency; Max Freq, maximum frequency; SD Freq, Standard deviation of frequency; Hz, Hertz.

The results considered for discussion were those with correlation ≥40%, comprising regular, good and excellent correlations. Thus, it was observed that as the voice detection threshold increases, i.e., worsens, the mean of *f*_0_ in the sustained vowel (*p* ≤ 0.001) and in spontaneous speech (*p* ≤ 0.005) increases (Table 3, Table 4, Table 5).

**Table 3:** Correlation of acoustic data of the sustained vowel with age, time of device use, voice detection threshold, hearing category and language category.

Parameter	Age		Time of use		VDT		Hearing category		Language category	
	Corr	*p*-Value	Corr	*p*-Value	Corr	*p*-Value	Corr	*p*-Value	Corr	*p*-Value
Mean *f*_0_ (Hz)	−30.70%	0.036	−17.90%	0.23	53.80%^a^	0.001^b^	−12.80%	0.391	−14.90%	0.317
SD *f*_0_ (Hz)	5.60%	0.708	19.70%	0.185	23.20%	0.174	−1.00%	0.947	−9.80%	0.513
v*f*_0_ (%)	13.80%	0.354	21.80%	0.141	2.90%	0.868	19.10%	0.198	0.20%	0.989
	27.10%	0.066	33.70%	0.021	−1.10%	0.949	26.50%	0.072	10.40%	0.486
vAmp (%)	9.10%	0.544	2.60%	0.862	−38.20%	0.022	−18.90%	0.202	−5.10%	0.734
Shim (%)	32.10%	0.028	38.10%	0.008	−11.60%	0.499	27.10%	0.065	15.80%	0.288
VTI (%)	−11.80%	0.43	3.50%	0.813	35.60%	0.033	−3.60%	0.81	1.00%	0.946
SPI (%)	16.20%	0.277	−3.80%	0.802	−12.20%	0.479	2.50%	0.867	4.50%	0.764
NHR (%)	18.60%	0.211	25.70%	0.081	−11.70%	0.496	25.70%	0.081	16.80%	0.259
DSH (%)	10.60%	0.48	8.90%	0.55	−11.30%	0.513	4.00%	0.792	−0.40%	0.979

VDT, voice detection threshold; Mean *f*_0_, mean of the fundamental frequency; SD *f*_0_, standard deviation of the fundamental frequency; v*f*_0_, coefficient of variation of *f*_0_, coefficient of amplitude variation; VTI, voice turbulence index; SPI, soft phonation index; HNR, harmonics-to-noise ratio; DSH, degree of sub-harmonics; Hz, Hertz.

a Corr ≥ 40%.

b *p* > 0.05.

**Table 4:** Correlation of acoustic data of sequential speech with age, time of device use, voice detection threshold, hearing category and language category.

Parameter	Age		Time of use		VDT		Hearing category		Language category	
	Corr	*p*-Value	Corr	*p*-Value	Corr	*p*-Value	Corr	*p*-Value	Corr	*p*-Value
Mean Freq (Hz)	−33.20%	0.026	−19.00%	0.212	26.00%	0.125	−20.20%	0.184	−17.10%	0.26
Extension (Hz)	−11.50%	0.451	−14.30%	0.349	−2.20%	0.898	0.90%	0.953	−4.20%	0.783
Min Freq (Hz)	−4.10%	0.787	8.30%	0.588	9.10%	0.598	−7.30%	0.634	3.80%	0.806
Max Freq (Hz)	−24.90%	0.099	−23.50%	0.12	10.40%	0.545	−3.80%	0.805	−12.50%	0.412
SD Freq (Hz)	−15.40%	0.319	−17.20%	0.264	7.60%	0.664	−9.70%	0.532	−23.60%	0.123
Extension semitones	−12.20%	0.425	−20.70%	0.173	2.40%	0.887	0.20%	0.989	−11.80%	0.438

VDT, voice detection threshold; Mean Freq, mean frequency; Min Freq, minimum frequency; Max Freq, maximum frequency; SD Freq, standard deviation of the frequency; Hz, Hertz.

**Table 5:** Correlation of acoustic data of spontaneous speech with age, time of device use, voice detection threshold, hearing category and language category.

Parameter	Age		Time of use		VDT		Hearing category		Language category	
	Corr	*p*-Value	Corr	*p*-Value	Corr	*p*-Value	Corr	*p*-Value	Corr	*p*-Value
Mean Freq (Hz)	−13.40%	0.366	−5.50%	0.711	44.90%^a^	0.005^b^	−3.80%	0.796	−2.90%	0.846
Extension (Hz)	2.70%	0.857	7.00%	0.635	7.90%	0.642	26.10%	0.073	16.60%	0.26
Min Freq (Hz)	−13.20%	0.371	−18.60%	0.206	13.30%	0.433	−20.60%	0.16	−16.10%	0.273
Max Freq (Hz)	−7.40%	0.615	−6.10%	0.681	19.70%	0.243	17.20%	0.241	8.40%	0.569
SD Freq (Hz)	−5.40%	0.715	8.10%	0.584	13.40%	0.429	19.60%	0.182	12.50%	0.398
Extension semitones	4.60%	0.755	10.00%	0.497	5.00%	0.767	25.00%	0.086	18.10%	0.218

VDT, voice detection threshold; Mean Freq, mean frequency; Min Freq, minimum frequency; Max Freq, maximum frequency; SD Freq, standard deviation frequency; Hz, Hertz.

a Corr ≥ 40%.

b *p*  >  0.05

## Discussion

Acoustic analysis quantifies the sound signal and provides enough documentation to outline the baseline of the individual's voice; the acoustic parameters of fundamental frequency and their disturbance indices, along with measurements of noise, have important clinical implications.^2^ Among all analyzed acoustic parameters, only the fundamental frequency showed a correlation with the assessed demographics and hearing aspects. Although moderate, this correlation stands out, considering the value of *p*.

Among many other features, the literature shows a trend in which hearing-impaired individuals have high pitched voices, with this being the perceptual correlation of high fundamental frequency, which is caused by the lack of auditory feedback of one's own voice, as the frequency control is affected when auditory functions are impaired.^4^, ^15^, ^16^

The fundamental frequency is given by the glottic cycle numbers over a time unit (seconds). Their determinant factors are the natural length of the vocal folds, stretching, mass vibration and the involved tension.^17^ A high fundamental frequency reflects poor laryngeal control, also demonstrated by elevation of the larynx, higher phonation effort and incapacity to control the tension of the vocal folds and subglottic pressure.^6^

It is possible that the lack of auditory feedback by decreased auditory thresholds causes an increase in tension during the glottic cycle^6^ and a lack of control of the laryngeal musculature, thus increasing the values of the fundamental frequency. However, while several authors report that hearing impaired individuals often have difficulties in controlling laryngeal function,^5^, ^6^, ^7^, ^11^, ^16^ no studies were found in literature evaluating the laryngeal behavior of the hearing-impaired, that could provide a clearer understanding of the characteristics and adjustments of the vocal tract that result in the vocal changes described in this population.

In this study, it was observed that both in the sustained vowel and in spontaneous speech, the participants with worse voice detection threshold had the highest frequency. As the hearing loss increases or a hearing threshold obtained with a hearing aid worsens, the voice parameters tend to be more deviated.^18^ However, results from other studies that analyzed the fundamental frequency of CI^5^, ^11^, ^19^, ^20^, ^21^, ^22^, ^23^ users are controversial. Thus, we stress the importance of knowing all aspects that may have an impact on the vocal characteristics of cochlear implant users.

A previous study^24^ found a correlation between fundamental frequency values and the result of a speech perception test. A correlation has also been found between frequency values and the time of rehabilitation and age at surgery.^11^ A study assessing the variability in fundamental frequency^4^ showed that as the auditory threshold worsens, the variability of the fundamental frequency increases due to a decrease in auditory signals for the monitoring of vocal production. Studies carried out among the elderly have also shown that as the tone thresholds worsen, *f*_0_ increases.^3^, ^25^

The impact of worsening hearing thresholds on the fundamental frequency measures has also been shown in studies that evaluated vocal production immediately after removing the auditory feedback provided by the CI.^15^

The other acoustic measures, including speech signal disturbances, utterance noise level and stability, did not correlate with the assessed data. Acoustic analysis of cochlear implant users demonstrated alterations of frequency and amplitude measures in short term with noise measurements within the normal range, and altered stability measures,^11^, ^20^, ^23^ with the results always associated with auditory feedback.

Understanding the physiological processes that contribute to vocal development in cochlear implanted children allows us to understand the speech production strategies in this population, attempting to establish goals for an adequate speech production.^26^ It is the auditory feedback that regulates the laryngeal musculature and postures of the vocal tract for an adequate phonation.^20^ Therefore, obtaining audiometric thresholds within the normal range for cochlear implant users is critical for vocal production control.

This study demonstrated the importance of understanding not only the vocal characteristics of cochlear implant users, but also with what factors these characteristics are correlated. This information is of great value for clinicians to increasingly ensure appropriate intervention regarding the vocal production of CI users.

## Conclusion

For the assessed cochlear implanted children, there was a correlation between the voice detection thresholds and the frequency values for the sustained vowel and also in spontaneous speech. That allows us to affirm that the worse the voice detection threshold is, the more high-pitched is the child's voice. Other acoustic parameters did not correlate with the other assessed variables.

## Conflicts of interest

The authors declare no conflicts of interest.
